# The Life and Letters of Dr. Samuel Hahnemann

**Published:** 1896-05

**Authors:** 


### BOOK NOTICES.

The Life and Letters of Dr. Samuel Hahnemann. By Thomas Lindsley Bradford, M. D. Philadelphia, Pa.: Bœricke & Tafel, 1895. 513 pages. Price in cloth, $2.50 net; by mail, $2.75. Half morocco, $3.50 net; by mail, $3.95.

The editor owes the author and the publishers alike an apology for the long delay in noticing this book. His excuse is that the story of Hahnemann's life has been told by Dr. Bradford in such fascinating style that he desired to read it through carefully before discussing its merits. Hence the failure to speak of it at an earlier date.

Dr. Bradford is very well known to the profession by his excellent book, *Homœopathic Bibliography of the United States.* This latter book shows an immense amount of research, as it is also the product of many years of laborious collecting of books and pamphlets devoted to Homœopathy, Dr. Bradford has thus established himself as an authority upon homœopathic literature. Therefore, when he appears before the profession as the writer of a life of Hahnemann, his work must receive much more than the usual attention, and must be accorded a most cordial reception because of the confidence inspired in him by his previous volume on the Bibliography of the School.

Nor are we disappointed when we examine the book. It is delightfully written in a style that compels attention and interest. Many points in Hahnemann's life not hitherto brought out, or else overlooked and forgotten, are here recorded. For instance, on page 316, it is related that Hahnemann was made honorary member of "The Medical Society of the City and County of New York," which was an Allopathic organization of the leading physicians of the old school in New York city! Hahnemann continued to be a member of this society for ten years, when his certificate of membership was withdrawn by the society about a week after his death! Of course the members could not have known of his death, as cable communication between Europe and this country had not been established. It is, however, an interesting item in the history of this great man.

All the statements in this biography are abundantly backed up with authorities, and at the end of the book is a list of all of Hahnemann's works.

A discussion of the *Chronic Diseases*, the work which, more than any other of Hahnemann's writings, has caused so much virulent controversy, is given with great clearness. It will be remembered that a review of the new edition of Hahnemann's *Chronic Diseases* was published in this journal in April. In that review mention was made of the inaccuracies of translation of the *Chronic Diseases* perpetrated by Dr. Hempel in the former English edition.

Dr. Bradford seems to have shared the opinions given in that review of the unreliability of Hempel's English rendering, for he has almost entirely ignored it in speaking of the *Chronic Diseases*, and appears rather to have depended upon a manuscript translation prepared by Dr. Korndœrfer. It is to be regretted this translation never saw the light. It probably will not, now that the version of Professor Tafel has been published.

In connection with this discussion of the *Chronic Diseases*, perhaps one of the ablest, as it is one of the most interesting parts of the book, is that in reference to Hahnemann's psora theory. A fine defense of Hahnemann is here given, an elaborate and vivid exposition of what he meant by psora, and a demonstration that Hahnemann *did* know of the existence of the itch insect. This last point has been persistently questioned, and Dr. Bradford, we think, has effectually settled it.

Nothing like this book has ever appeared concerning the master of the healing art, and nothing too much in praise of it can be said of it by any reviewer. Nothing commensurate with its merits has been said in this notice. To do it justice would require more space and more time than is at the command of the editor, and so this abbreviated commentary must serve to cordially recommend Dr. Bradford's fine effort to the attentive perusal of all who venerate Hahnemann.

